# Guardians or Gateways? The Intricate Relationship Between Plant Cell Walls and Pathogenic *Xanthomonas*

**DOI:** 10.1111/mpp.70292

**Published:** 2026-06-19

**Authors:** Charlotte Gaudin, Marie‐Agnès Jacques, Nicolas W. G. Chen

**Affiliations:** ^1^ Univ Angers, Institut Agro, INRAE, IRHS, SFR QUASAV Angers France

## Abstract

Plants have evolved a complex cell wall (CW) providing support and protection against environmental constraints, including constant attacks from pests and pathogens. Indeed, the plant CW serves as both a physical barrier and a sophisticated monitoring and signalling system, making it a central component of plant immunity. The *Xanthomonas* genus encompasses a large diversity of plant‐pathogenic bacteria that, together, can infect a wide range of more than 400 plant species including monocots and dicots. Remarkably, *Xanthomonas* species are subdivided into highly specialized pathovars infecting a narrow range of plant species and/or tissues, each possessing a distinct CW structure and composition. This diversity makes *Xanthomonas* a perfect case for studying the interactions between bacterial pathogens and the plant CW. In this review, we provide an overview of the intricate interactions between *Xanthomonas* and the plant CW during the infection process. During infection, *Xanthomonas* degrades the plant CW both directly by using CW degrading enzymes and indirectly by reprogramming the plant transcriptome to enhance the expression of plant CW modifying enzymes. This degradation of the plant CW plays a central role during infection. On the plant side, it triggers immune responses, while on the *Xanthomonas* side, it facilitates bacterial invasion and access to nutrients, and activates a signalling hub that primes the pathogen for host colonization.

## Graphical abstract

An overview of the intricate interactions between *Xanthomonas* and the plant cell wall during the infection process.

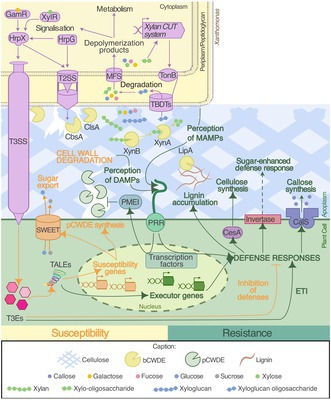

## 1 Introduction

The plant cell wall (CW) is a complex network of polysaccharides, proteins, and other polymers such as lignin or suberin, forming a highly organized matrix that contributes to the structuring of plant tissues and organs (Albersheim et al. 2010; Anderson and Kieber 2020; Wolf 2022). Beyond its architectural role, the CW is a dynamic structure that actively participates in fundamental physiological processes such as cell expansion and division, water transport, and cell‐to‐cell communication, all of which are essential for plant growth and development (Cosgrove 2005; Houston et al. 2016). Moreover, the CW serves as the primary interface between plant cells and their external environment, mediating critical interactions with both abiotic and biotic factors. It has been extensively documented that, during microbial infection, pathogens often target the plant CW to breach plant defences, while plants reinforce or modify their CW to resist invasion, making it a strategic point for both the attacker and the defender (Hématy et al. 2009; Houston et al. 2016; Hückelhoven 2007). Indeed, together with the cuticle, the CW is the first layer of plant defences against pathogen invasion, acting both as a physical barrier and as a monitoring system (Bacete et al. 2018; Bhandari et al. 2025; Houston et al. 2016; Molina et al. 2024; Underwood 2012; Wan et al. 2021). However, the CW can be degraded by CW degrading enzymes (CWDEs) and exploited by pathogens to access nutrients and promote infection (Bellincampi et al. 2014; Cantu et al. 2008; Walton 1994).

The *Xanthomonas* genus groups gram‐negative, rod‐shaped, obligate aerobic bacteria with a specialized lifestyle on plants. *Xanthomonas* may have a pathogenic or a commensal lifestyle (Pena et al. 2024). At the genus level, pathogenic *Xanthomonas* are responsible for diseases affecting nearly 400 plant species, most of which are crops of high economic value such as rice, pepper, tomato, cabbage, legumes, cotton, as well as woody species such as citrus and walnut trees (An et al. 2020; Hayward 1993; Jacques et al. 2016). Because of their impact on crop production, pathogenic *Xanthomonas* are more studied than commensals. *Xanthomonas* species are subdivided into pathovars, which are infra‐subspecific groups of strains having the same symptomatology and tissue specificity on the same host range (Young et al. 1978). Altogether, *Xanthomonas* cause a large array of symptoms including water‐soaked spots evolving into necrotic areas on leaves, dieback, wilt, hypertrophy, hyperplasia, rot, and cankers (Jacques et al. 2016; Rudolph 1993). This diversity of symptoms reflects different life cycles among the various pathovars of *Xanthomonas*. In most cases, infection of aerial plant organs starts with an epiphytic phase, where *Xanthomonas* bacteria attach themselves to the plant's external surface using their own surface polysaccharides, adhesion proteins, and type IV pilus, followed by biofilm formation (An et al. 2020; Jacques et al. 2016). Then, bacteria enter through wounds or natural openings such as stomata, hydathodes, lenticels, and nectaries, leading to distinct types of colonization processes depending on their host or tissue specificity (Jacques et al. 2016).

## 2 Breaking the Plant Cell Wall

### 2.1 Bacterial CWDEs: A First Step Towards Accessing the Host Cell

**FIGURE 1:**
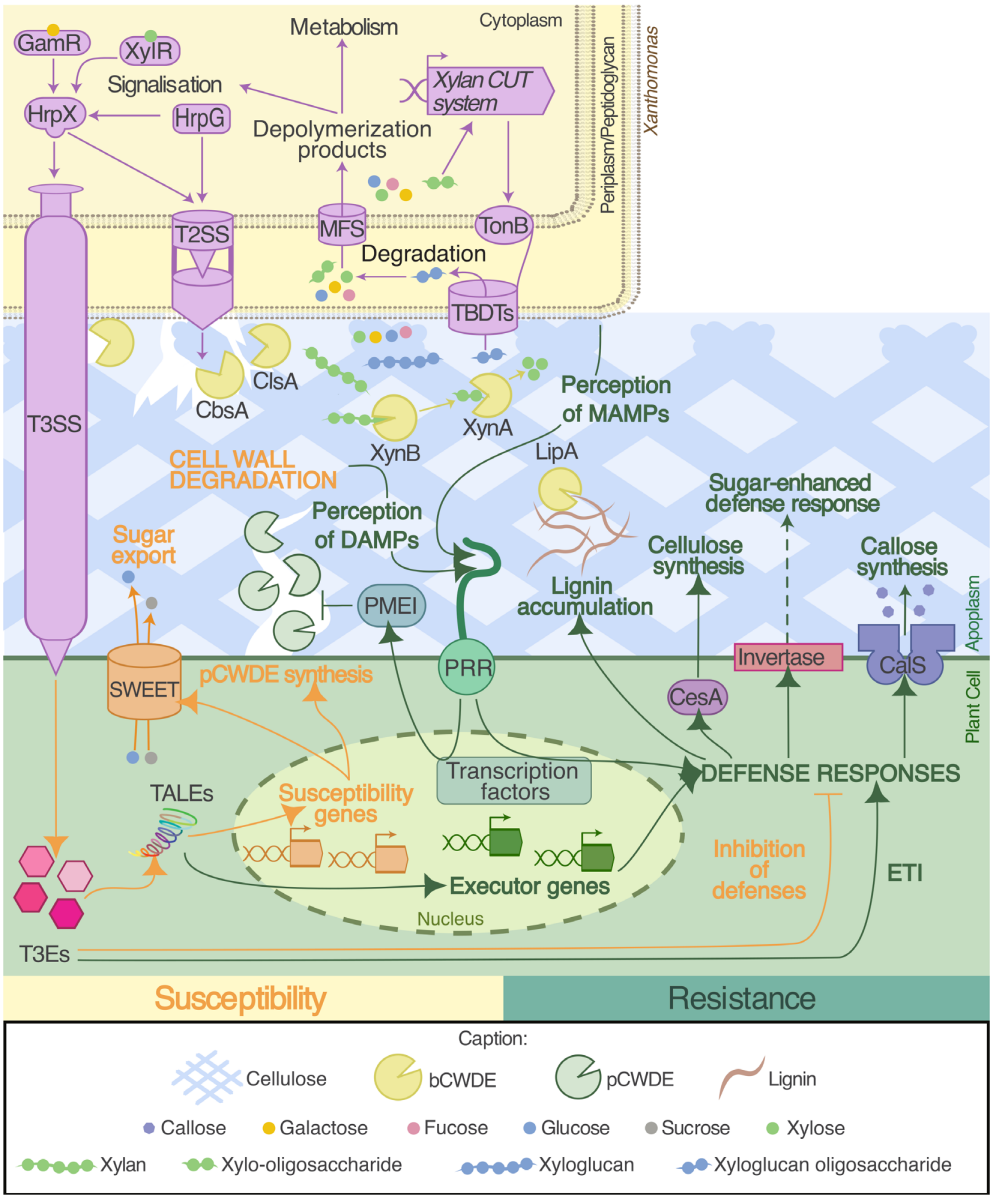
Key interaction pathways between *Xanthomonas* and the plant cell wall. Bacterial and plant cell wall‐degrading enzymes (bCWDEs and pCWDEs, respectively) facilitate the hydrolysis of structural polysaccharides of the plant cell wall (CW) such as xylan and cellulose, releasing degradation products contributing to *Xanthomonas* pathogenicity. The carbohydrate utilization system (CUT), mediated by the TonB‐dependent transporters (TBDTs) and major facilitator superfamily (MFS) transporters, processes these xylo‐oligosaccharide‐ and other polysaccharide‐derived fragments into the bacterial cytoplasm. Within the bacteria, these plant CW degradation products function both as nutritional sources and as signalling molecules, activating the pathogenicity‐associated Type II and Type III secretion systems (T2SS and T3SS, respectively) through regulators including XylR, GamR, HrpG, and HrpX. Activation of the T2SS leads to increased secretion of bCWDEs in the apoplast, while the T3SS injects type III effectors (T3Es) directly inside the plant cell. T3Es participate in susceptibility, possibly by inhibiting plant defences. Among T3Es, transcription activator‐like effectors (TALEs) are able to hijack the plant transcriptome, leading to increased production of pCWDEs or sucrose export via SWEET transporters. Similarly to microbe‐associated molecular patterns (MAMPs), plant CW degradation products can be perceived as damage‐associated molecular patterns (DAMPs) by pattern recognition receptors (PRRs), leading to plant defence activation. T3Es can also trigger plant defences through effector‐triggered immunity (ETI). Plant defence responses include the production of pCWDE inhibitors such as the pectin methylesterase inhibitor (PMEI), as well as plant reinforcement functions such as the accumulation of lignin and the production of callose and cellulose by callose synthase (CalS) and cellulose synthase (CesA), respectively.

To interact with their neighbouring environment, *Xanthomonas* employ type I to type VI secretion systems (T1SS to T6SS) able to secrete effectors having diverse functions and to deliver them either extracellularly or directly into target cells (Alvarez‐Martinez et al. 2021). Among those, pathogenic *Xanthomonas* bacteria use bacterial CWDEs (bCWDEs) to degrade the plant CW after entering the plant tissues (Figure 1). These enzymes belong to different functional families including cellulases, xylanases, pectinases, glucanases and esterases. They are secreted via the *xps* type II secretion system (T2SS) or alternatively by outer membrane vesicules (Solé et al. 2015; Tayi et al. 2016a, 2016b; Wang et al. 2008). The repertoire of bCWDEs may play an important role in determining the *Xanthomonas* lifestyle. Indeed, genomic analyses demonstrated clear distinctions in the repertoire of carbohydrate‐active enzymes between commensals and pathogens (Cesbron et al. 2015; Pena et al. 2024). This observation suggests that specific bCWDE combinations enable a commensal lifestyle, while others are essential for the pathogenicity of *Xanthomonas*. The importance of bCWDEs for pathogenicity has been demonstrated by functional studies. For example, mutation of the *xps* T2SS or of individual extracellular endoglucanases in *X. citri* pv. *citri* delays the onset of canker symptoms in citrus plants (Xia et al. 2016). Similarly in *X. vasicola* pv. *holcicola*, the glycosyl hydrolase GH5, the β‐glucosidase Bgl and the cellobiosidase Cbh each participate in increased bacterial fitness during *Sorghum* infection (Wang et al. 2025).

The bCWDEs from rice bacterial leaf blight agent *X. oryzae* pv. *oryzae* have been extensively studied for their role in virulence (Tayi et al. 2016a, 2016b, 2018). Several bCWDEs have a major effect on the disease outcome. This is the case of the endo‐1,4‐β‐D‐glucanase eglXoB, which is involved in cellulose hydrolysis and contributes to virulence and *in planta* growth of *X. oryzae* pv. *oryzae* (Hu et al. 2007). Additional bCWDEs play a more limited role in virulence. For example, ClsA is a cellulase that has both exo‐ and endoglucanase activities (Jha et al. 2007), while LipA is an esterase proposed to cleave alkyl ester cross‐links within polysaccharide chains of the lignin, possibly increasing accessibility for other degrading enzymes (Aparna et al. 2009). Individual knockout mutations of *lipA* and *clsA* partially reduce the virulence of *X. oryzae* pv. *oryzae* in rice, while the double mutant is severely compromised (Jha et al. 2007). This suggests that LipA and ClsA act complementarily to each other for full virulence. Similarly, the xylanase XynB was shown to have a partial effect on aggressiveness, complementary to LipA (Rajeshwari et al. 2005). Mutations in other genes such as *pglA*, *pmt*, *pel* and *pelL* encoding pectinolytic enzymes had little or no effect on the aggressiveness of *X. oryzae* pv. *oryzae* (Tayi et al. 2016b). This suggests that some bCWDEs may not contribute to pathogenicity, unless functional redundancy masked their individual effect. Answering this question would require analysing strains mutated in multiple bCWDE‐encoding genes.

Degradation products from bCWDEs activity, such as xyloglucan and xylans, are imported into *Xanthomonas* cells via TonB‐dependent transporters, then further depolymerized by multiple carbohydrate‐degrading enzymes in the periplasm and cytoplasm, leading to xylo‐oligosaccharides and sugar monomers such as xylose, galactose, glucose, and fucose, whose metabolization enhances bacterial growth (Chow et al. 2015; Déjean et al. 2013; Santos et al. 2014; Vieira et al. 2021). The whole process is coordinated by operons of co‐regulated genes, also known as carbohydrate utilization with TonB‐dependent transporter (CUT) loci (Blanvillain et al. 2007; Déjean et al. 2013). In *X. campestris* pv. *campestris* and *X. oryzae* pv. *oryzae*, short xylo‐oligosaccharides and xylose strongly induce xylan CUT loci, which are otherwise repressed by the XylR LacI‐type repressor (Déjean et al. 2013; Ikawa et al. 2018; Santos et al. 2014), and a similar regulation has been proposed for *X. citri* pv. *citri* (Vieira et al. 2021). This conserved mechanism underscores how cell wall‐derived sugars, processed by the CUT systems, modulate nutrient uptake in *Xanthomonas*.

### 2.2 Putative Role of bCWDEs in Host Specificity

Understanding host specificity and adaptation in *Xanthomonas* is an important issue that was raised long ago but has so far received few answers (An et al. 2020; Dye 1958; Jacques et al. 2016; Timilsina et al. 2020). Based on comparative genomics of strains from the *X. axonopodis* species complex with distinct host ranges, type III effectors (T3Es), sensors, and adhesins were described as potential determinants of host specificity, as the repertoire of these factors is often correlated with the ability to infect specific plant species (Constantin et al. 2016; Hajri et al. 2009; Mhedbi‐Hajri et al. 2011).

Additional studies have highlighted the potential role of bCWDEs in shaping the host range and pathogenicity of *Xanthomonas* pathovars. An early example emerged from comparative genomics between *X. citri* pv. *citri* and *X. campestris* pv. *c* *ampestris*, responsible for citrus canker on *Citrus* spp. and black rot of Brassicaceae, respectively (da Silva et al. 2002). Notably, *X. campestris* pv. *campestris* harbours more bCWDE‐encoding genes than *X. citri* pv. *citri*, a difference that may directly reflect their distinct symptomatology. While *X. citri* pv. *citri* causes localized watersoaking and necrosis, *X. campestris* pv. *campestris* leads to extensive tissue maceration. This putative link between the symptom severity and the bCWDE arsenal is further supported by differences within the *rpf* gene cluster, which regulates the transcription of bCWDE‐encoding genes (Dow et al. 2000). Specifically, the *rpfI* gene, which enhances the expression of proteases and endoglucanases in *X. campestris* pv. *campestris*, is absent in *X. citri* pv. *citri* (da Silva et al. 2002).

Beyond symptomatology, the correlation between host specificity and bCWDE repertoires may reflect host‐related factors other than symptomatology, such as the ability to infect a specific range of host plants. For example, different sets of bCWDEs were described between *X. citri* pv. *citri* and *X. arboricola* pv. *pruni*, although these pathovars produce similar symptoms on *Citrus* spp. and *Prunus* spp., respectively (Garita‐Cambronero et al. 2019). Similarly, comparative genomics of four *Xanthomonas* pathovars infecting tomato and pepper revealed that differences in host range and virulence are associated with variations in bCWDE repertoires, both in terms of gene presence/absence and genomic arrangement patterns (Potnis et al. 2011). In the strawberry pathogen *X. fragariae* , the reduced number of bCWDE‐encoding genes and the absence of xylan CUT loci suggest that the pathogen has limited CW degradation capabilities (Vandroemme et al. 2013). The authors suggest that limited damage intensity to the CW could prevent potential release of toxic compounds from the phenolic‐rich strawberry tissues.

### 2.3 Role of bCWDEs in Tissue Specificity

The pathovar concept does not solely rely on host range but also includes tissue specificity (Dye 1980; Jacques et al. 2016; Lu et al. 2008). Indeed, colonizing vascular or parenchymatic tissues might represent different challenges for the bacteria due to differences in composition between the xylan‐rich dead cell walls of the xylem and the living cells of the parenchyma, which are rich in proteinaceous compounds (Zou et al. 2012). Some pathovars enter preferentially through stomata, leading to invasion of the mesophyll, while others enter through hydathodes and proliferate in the xylem vessels. A few pathovars are able to colonize both the mesophyll and the vascular elements, which is the case of common bean pathogens *X. citri* pv. *fuscans* and *X. phaseoli* pv. *phaseoli* (Chen et al. 2021; Mijatović et al. 2021).

Tissue specificity in *Xanthomonas* is best exemplified by the two rice pathogens, *X. oryzae* pv. *oryzae* and *X. oryzae* pv. *oryzicola*, which respectively adopt a vascular and parenchymatous lifestyle on the same host plant (Niño‐Liu et al. 2006). Although both *X. oryzae* pv. *oryzae* and *X. oryzae* pv. *oryzicola* can grow within the intercellular spaces between mesophyll cells, only *X. oryzae* pv. *oryzicola* demonstrates the ability to propagate within extracellular spaces, resulting in leaf streak lesions (Cao et al. 2020). In comparison to *X. oryzae* pv. *oryzae*, *X. oryzae* pv. *oryzicola* shows a greater ability to degrade the mesophyll CW and cellulose filter papers (Cao et al. 2020). Moreover, compared to vascular tissues, mesophyll tissues comprise embedded proteinaceous compounds whose degradation by *Xanthomonas* may require specific protease activity. Interestingly, the C‐terminal residues of the extracellular protease EcpA differ between *X. oryzae* pv. *oryzae* and *X. oryzae* pv. *oryzicola*, making the protein inactive in *X. oryzae* pv. *oryzae* while it participates in the aggressiveness of *X. oryzae* pv. *oryzicola* (Zou et al. 2012). The ability of purified *X. oryzae* pv. *oryzicola*'s EcpA to elicit bacterial leaf streak symptoms supports its role as a mesophyll‐specific virulence factor (Zou et al. 2012).

Combination of genomics and functional analyses highlighted the cellobiosidase CbsA as a key factor to vascular *Xanthomonas* lifestyle (Gluck‐Thaler et al. 2020). CbsA hydrolyses glycosidic bonds and, through its exoglucanase activity, can processively hydrolyse crystalline cellulose (Kumar et al. 2012; Tayi et al. 2018), enabling efficient xylem CW hydrolysis and full virulence of *X. oryzae* pv. *oryzae* (Jha et al. 2007; Tayi et al. 2018). Ectopic *cbsA* expression in nonvascular *X. perforans* pv. *undulosa* enables vascular symptom development, while its knockout in vascular *X. perforans* pv. *translucens* increases mesophyll lesions without compromising vascular symptoms (Gluck‐Thaler et al. 2020). Patterns of *cbsA* presence/absence and horizontal transfer suggest that dynamic gains and losses of *cbsA* have shaped the evolution of vascular versus non‐vascular lifestyles in *Xanthomonas*.

The vascular *X. campestris* pv. *campestris* enters preferentially through hydathodes, organs present at the leaf margin of vascular plants that are dedicated to guttation through stomata‐like pores (Bellenot et al. 2022; Cerutti et al. 2019). Beneath the pores, hydathodes form an aqueous extracellular cavity constituted by a specific tissue of loosely connected cells with thin, fibrillar CWs, which display specific immune responses (Cerutti et al. 2017; Paauw et al. 2023; Pfeilmeier et al. 2025). In *Arabidopsis*, a functional xps‐T2SS is not necessary for the bacteria to colonize the xylem vessels, but is essential to pass from the hydathode to the xylem, and from the midvein to lateral veins (Paauw et al. 2024). Moreover, four bCWDEs: CbsA, LipA, a cellulase and a β‐xylosidase, respectively, contribute to the vascular spread of the bacteria, with CbsA playing a predominant role (Paauw et al. 2024). This further highlights the role of bCWDEs in tissue specificity.

In conclusion, the repertoire and regulation of bCWDEs, along with T3Es, sensors, and adhesins, provide a robust molecular framework for understanding host and tissue specificity among *Xanthomonas* pathovars.

## 3 Transcriptomic Hijack at the Plant Cell Wall

### 3.1 Plant CWDEs: A Second Wave of CW Degradation to Get the Job Done

On the plant side, transcriptomic studies highlighted differential expression of CW‐associated genes upon infection by different *Xanthomonas* pathovars, often with contrasted patterns between resistant and susceptible plants. In a compatible context, a general observed pattern is the overexpression of genes encoding plant CWDEs (pCWDEs). Indeed, in different plants such as rice, common bean or sweet orange, successful infection by *Xanthomonas* is linked to upregulation of genes encoding enzymes and proteins involved in cell expansion, cell division and CW loosening, such as expansins and pectate lyases (Cernadas et al. 2008; Foucher et al. 2020; Liao et al. 2019). These observations suggest that *Xanthomonas* bacteria are able to trigger the induction of pCWDE‐encoding genes in susceptible plants, which, in addition to their own bCWDEs, could further improve their ability to degrade the plant CW (Figure 1).

### 3.2 TALEs to Manipulate the Plant CW

Transcription activator‐like effectors (TALE) are *Xanthomonas*‐specific T3Es specialized in hijacking plant transcriptomes by inducing specific target genes (Perez‐Quintero and Szurek 2019). TALEs mimic eukaryotic transcription factors by inducing gene expression upon binding the host DNA through a central domain composed of tandem repeats that confer their specificity towards a target DNA sequence (Boch et al. 2009; Moscou and Bogdanove 2009). TALE targets, also called effector‐binding elements (EBEs), are usually located within promoters of susceptibility (*S*) genes whose induction leads to enhanced symptoms and/or bacterial proliferation (Boch et al. 2014).

One of the most‐studied classes of *S* genes encode sugar transmembrane transporters from the SWEET family (Chen 2014; Mondal et al. 2021; Perez‐Quintero and Szurek 2019). In particular, the clade III SWEET subfamily constitutes a major class of *S* genes in plants, most extensively characterized in rice (Antony et al. 2010; Römer et al. 2010; Streubel et al. 2013; Yang et al. 2006; Yu et al. 2011), cassava (Cohn et al. 2014) and cotton (Mormile et al. 2025). Clade III *SWEETs* are involved in the release of sucrose in the apoplast (Chen et al. 2010; Chen et al. 2012). Thus, overexpression of *SWEETs* by TALEs could favour *Xanthomonas* proliferation by serving as a direct source of carbon, although actual catabolism of secreted sucrose by the bacteria is yet to be proven (Cohn et al. 2014; Zhou et al. 2015). Alternatively, increased apoplastic sucrose concentration could also interfere with the apoplast water potential, normal membrane function or sucrose signalling pathways (Huang et al. 2016; Mormile et al. 2025).

Another common class of *S* genes encode transcription factors, which, in turn, induce the overexpression of secondary targets (Boch et al. 2014). In most cases, secondary targets correspond to pCWDEs, thereby contributing to the development of symptoms. In tomato, the TALE AvrHah1 from *X. gardneri* induces the transcription of two bHLH transcription factors, which in turn upregulate a pectate lyase and a pectinesterase, respectively (Schwartz et al. 2017). This induction of pCWDEs leads to pectin modifications and to the development of water‐soaked lesions involving CW loosening and increased water movements. In pepper, AvrBs3 from *X. euvesicatoria* pv. *vesicatoria* triggers hypertrophy of mesophyll tissues by targeting the *upa20* gene, which encodes a bHLH transcription factor (Kay et al. 2007; Marois et al. 2002). This leads to the induction of auxin‐responsive and expansin‐like genes, both of which are known to promote cell enlargement (Marois et al. 2002). In *Citrus* spp., two TALEs, PthA4 and PthAw, from *X. citri* pv. *citri* both activate *CsLOB1*, a member of the Lateral Organ Boundaries (LOB) family of transcription factors, thereby promoting pustule formation (Hu et al. 2014). Overexpression of *CsLOB1* by PthA4 is linked to the upregulation of pCWDEs associated with cell expansion, CW degradation, modification, and cell division, such as expansins and pectate lyases in both sweet orange and Meiwa kumquat (Hu et al. 2014; Teper et al. 2020). In citrus leaves and fruits, this transcriptomic hijack leads to the activation of the fruit ripening pathway, which releases soluble carbohydrates including xylose into the surrounding tissue (Phan et al. 2025). Xylose then induces the xylan CUT system, including a T2SS‐secreted xylanase, which amplifies CW degradation in a feedforward loop. This hijacking of the fruit ripening pathway demonstrates the ability of TALEs to trick the plant cell into producing nutrients for *Xanthomonas* (Kvitko and Jacobs 2025). By inducing CW‐related regulators, TALEs synergize with pCWDEs, thereby shaping disease development. Therefore, hijacking pCWDEs emerges as a key strategy by which some *Xanthomonas* pathovars enhance CW loosening to optimize infection (Figure 1).

## 4 Resisting the Invasion: Another Brick in the Wall

### 4.1 Defence Activation by CWDEs: A Double‐Edged Sword

The activity of both bCWDEs and pCWDEs results in the production of degradation compounds (Figure 1), some of which the plant immune system can perceive as damage‐associated molecular patterns (DAMPs). These DAMPs serve as a universal danger signal for plants (Dora et al. 2022; Hou et al. 2019; Molina et al. 2024; Tanaka and Heil 2021). Extracellular perception of DAMPs is due to transmembrane pattern recognition receptors (PRRs), which can also directly detect pathogens through the perception of conserved molecules called microbe‐associated molecular patterns (MAMPs). Detection of either DAMPs or MAMPs by PRRs leads to the activation of pattern‐triggered immunity (PTI) responses (Bacete et al. 2018; DeFalco and Zipfel 2021).

For example, *Capsicum annuum* CW degradation by *X. campestris* pv. *campestris* leads to the production of oligosaccharides with DAMP activity (Vorhölter et al. 2012). The process appears to involve trans‐envelope signalling by TonB‐dependent receptor. In Meiwa kumquat, indirect upregulation of pCWDEs by the TALE PthA4 from *X. citri* pv. *citri* appears to play a dual role, leading to enhanced symptoms of hypertrophy and hyperplasia followed by a hypersensitive response‐like defence phenotype (Teper et al. 2020). One plausible explanation for this might be that pCWDEs contribute both to the virulence of *Xanthomonas* by degrading the CW and to the activation of plant defences by producing DAMPs. Similarly, lowered plant defence responses were observed in various pathosystems after inoculation of *xps‐*T2SS or bCWDE mutants (Jha et al. 2007; Szczesny et al. 2010; Tayi et al. 2016a; Teper et al. 2020). Together, these observations suggest that DAMP‐mediated PTI may stem from the recognition of CW damage caused by the activity of pCWDEs, bCWDEs, or a combination of both (Teper et al. 2020).

### 4.2 Plant Defences Involve Both CW Fortification and the Suppression of CW Degradation

In contrast to susceptibility, plant resistance to *Xanthomonas* is linked to downregulation of pCWDE‐encoding genes, together with upregulation of genes whose function putatively counteracts CW degradation and/or strengthens the plant CW. This is the case in sweet orange, Meiwa kumquat, rice and common bean, where *Xanthomonas*‐triggered defence is characterized by the upregulation of genes linked to CW strengthening, lignification, and downregulation of pCWDEs (Cernadas et al. 2008; Foucher et al. 2020; Girija et al. 2017; Khalaf et al. 2011; Wang et al. 2019; Yu et al. 2014).

As shown for plant resistance to various pathogens, resistance to *Xanthomonas* usually involves CW reinforcement, including cellulose synthesis, callose and/or lignin deposits. For instance, PRR‐encoding genes *Xa4* and *Xa21* or *Executor* genes *Xa7* and *Xa10* were shown to promote cellulose synthesis and/or lignin accumulation in rice (He et al. 2024; Hu et al. 2017; Reimers and Leach 1991; Shamsunnaher et al. 2020). In cassava, resistance to *X. phaseoli* pv. *manihotis* is associated with enhanced lignification and callose deposition; in addition, pectin and/or lignin accumulate to plug the xylem, resulting in bacterial confinement and limitation of secondary spreads (Kpémoua et al. 1996). In lemon ( *Citrus* x *limon* ), RNAi knockdown of a callose synthase increases susceptibility to *X. citri* pv. *citri*, indicating a significant role of callose biosynthesis for resistance (Enrique et al. 2011). CW reinforcement appears to be a common response to both pathogenic and nonpathogenic *Xanthomonas*, as exemplified by the accumulation of callose deposits after inoculation of common bean leaves with a *hrpA* mutant of *X. vesicatoria* or a saprophytic strain of *X. campestris* (Brown et al. 1998). CW integrity during defence responses can be further maintained by pCWDE inhibitors, as it is the case for the pectin methylesterase inhibiting enzyme CaPMEI1 in pepper (An et al. 2008) or for the polygalacturonase‐inhibiting protein OsPGIP1 in rice (Wu et al. 2019).

Several transcription factor families can orchestrate CW reinforcements during resistance. For instance, *OsMYB63*‐overexpressing and *OsWRKY53*‐knockout rice lines present thickened CWs and higher resistance to *X. oryzae* pv. *oryzae* (Xie et al. 2021). Mechanistically, OsMYB63 promotes cellulose deposition by upregulating cellulose synthase genes, while *OsWRKY53* promotes susceptibility by directly suppressing the expression of *OsMYB63* (Xie et al. 2021). In common bean, overexpression of transcription factor *PvOFP7* contributes to the resistance to *Xanthomonas* and is linked to the upregulation of genes involved in cellulose, hemicellulose, pectin, xylan, and xyloglucan biosynthesis (Foucher et al. 2020; Gaudin et al. 2025).

Altogether, resistance to *Xanthomonas* appears to be consistently associated with CW reinforcement through multiple structural modifications and suppression of CW degradation. These structural reinforcements likely contribute to limiting bacterial spread and damage within the host tissues.

## 5 Interplay Between Suppression of Defences and Cell Wall Degradation

### 5.1 Inhibition of CW‐Related Defences

To circumvent the plant's defences, *Xanthomonas* bacteria employ different strategies, including the production of extracellular compounds such as the exopolysaccharide xanthan or the cyclic β‐(1,2)‐glucan, which are both able to suppress callose deposition in *Nicotiana benthamiana* and *Arabidopsis* (Rigano et al. 2007; Yun et al. 2006). Plant resistance can also be bypassed by the action of bacterial T3Es, which are secreted in the host cell via the T3SS and appear to be tightly linked to the suppression of CW‐based defences (Brown et al. 1995; White et al. 2009). For example, XopJ from *X. euvesicatoria* pv. *vesicatoria* targets the plasma membrane of host cells and inhibits CW‐based defences in solanaceous plants (Bartetzko et al. 2009). Similarly, XopB from *X. euvesicatoria* pv. *vesicatoria* interferes with a CW‐bound invertase to suppress PTI (Sonnewald et al. 2012). In rice, the action of the bCWDEs ClsA, CbsA, and Xyn plays a critical role in inducing defence responses during infection by *X. oryzae* pv. *oryzae* (Tayi et al. 2016a). These three enzymes lead to the induction of PTI, possibly through the production of soluble DAMPs, although the exact nature of these DAMPs is still to be determined. PTI is then suppressed by T3Es produced by *X. oryzae* pv. *oryzae* for successful infection (Jha et al. 2007). Similarly, XopN, XopQ, XopX, and XopZ are T3Es capable of suppressing rice innate immune responses induced by the bacterial esterase LipA (Sinha et al. 2013). In cassava, XopAE is able to suppress basal defences such as callose deposition and production of reactive oxygen species (Gómez De La Cruz et al. 2025).

#### 5.1.1 Degrading the CW to Facilitate T3SS Progression Towards the Plant Cell

To access the plant cell, the T3SS machinery has to pass the CW, which constitutes a physical barrier against pathogens (Beliën et al. 2006). Therefore, it was hypothesised that CW degradation by *xps*‐T2SS‐secreted bCWDE could facilitate the assembly of the T3SS towards the plant cell (Szczesny et al. 2010). However, the possibility of PTI induction by DAMPs raises the necessity to coordinate these two components of the *Xanthomonas* arsenal. In *X. citri* pv. *citri*, XynB was shown to work in tandem with the exo‐oligoxylanase XynA (Santos et al. 2014). XynB breaks down xylan into xylo‐oligosaccharides, which could ease nutrient access and bacterial T3E translocation, while XynA further degrades xylo‐oligosaccharides into xylose units, which could contribute to the inhibition of oligosaccharide‐induced immune responses (Santos et al. 2014). Therefore, breaking plant barriers by using bCWDEs appears to serve a dual purpose for *Xanthomonas*, not only for aiding the bacteria to progress into the plant tissues by dismantling the CWs and freeing new sources of nutrients, but also to pave the way for the T3SS machinery to access the plant cell and disrupt plant defence responses. In this regard, CW degradation stands as a pivotal stage for both *Xanthomonas* and plants.

#### 5.1.2 Coordination Between the T2SS and the T3SS

The intricate regulatory interplay between the *Xanthomonas* T3SS and *xps* T2SS is exemplified by their coordinated response to plant CW degradation products. In *X. citri* pv. *citri* and *X. oryzae* pv. *oryzae*, these products are depolymerized into galactose, which activates the T3SS through the master regulator HrpX (Rashid et al. 2016; Vieira et al. 2021). Similarly, xylose accumulation enhances virulence by increasing the expression of *hrp* regulatory genes (Alexandrino et al. 2023).

Conversely, the expression of T2SS genes and bCWDEs can be activated by the two master regulators of the T3SS HrpX and HrpG (Szczesny et al. 2010; Wang et al. 2008). Thus, the *xps*‐T2SS and T3SS appear to be part of a coordinated regulatory network, enabling mutual enhancement during the infection process. Taken together, these findings highlight that CW degradation is tightly intertwined with T2SS and T3SS function. bCWDE activity not only facilitates tissue colonization but also produces metabolites and signals that modulate T3SS gene expression and effector translocation. This coordinated strategy enables *Xanthomonas* to disarm CW‐based defences and establish a favourable environment ahead of immune recognition.

## 6 Conclusions and Perspectives

Many examples highlight the important role of the plant CW during pathogen colonization of the plant, and *Xanthomonas* makes no exception. However, *Xanthomonas* bacteria have their own way to use the plant CW, especially through the use of TALEs. In addition, host and tissue specificity appear to be tightly linked to the plant CW composition, but the molecular bases of these specificities are still not completely clear, especially regarding the regulation of plant immunity to *Xanthomonas*. For example, the MKP1‐MAPK cascade has been demonstrated to positively regulate vascular versus mesophyll resistance by activating lignin biosynthesis through the repression of MYB4 transcription factors (Lin et al. 2022). This sheds light on how a single signalling cascade can drive distinct immune outcomes against pathogens with varying lifestyles, but it is still unclear how this cascade is triggered by *Xanthomonas*. Conversely, the resistance protein SUT1 has been demonstrated to confer early resistance to *X. campestris* pv. *campestris* within the hydathodes specifically, and not within mesophyll or vascular tissues, but the signalling cascade leading to this resistance remains to be described (Taks et al. 2025). Additional components involving the plant CW for plant defences are still to be explored. For example, recent work in lemon revealed that apoplastic acidification is involved in the resistance to *X. citri* pv. *citri* (Ye et al. 2025), while pH acidification can alter CW properties (Phyo et al. 2019). Exploring the potential interplay between these mechanisms could provide new insights into plant defence strategies against *Xanthomonas*.

## Author Contributions

**Marie‐Agnès Jacques:** conceptualization, writing – review and editing. **Nicolas W. G. Chen:** conceptualization, writing – original draft, writing – review and editing, supervision, project administration. **Charlotte Gaudin:** conceptualization, writing – original draft, writing – review and editing, visualization.

## Conflicts of Interest

The authors declare no conflicts of interest.

## Data Availability

Data sharing is not applicable to this article as no new data were created.
